# Are We There Yet? - A Systematic Literature Review on Chatbots in Education

**DOI:** 10.3389/frai.2021.654924

**Published:** 2021-07-15

**Authors:** Sebastian Wollny, Jan Schneider, Daniele Di Mitri, Joshua Weidlich, Marc Rittberger, Hendrik Drachsler

**Affiliations:** ^1^ Information Center for Education, DIPF | Leibniz Institute for Research and Information in Education, Frankfurt am Main, Germany; ^2^ Educational Science Faculty, Open University of the Netherlands, Heerlen, Netherlands; ^3^ Computer Science Faculty, Goethe University, Frankfurt am Main, Germany

**Keywords:** chatbots, education, literature review, pedagogical roles, domains

## Abstract

Chatbots are a promising technology with the potential to enhance workplaces and everyday life. In terms of scalability and accessibility, they also offer unique possibilities as communication and information tools for digital learning. In this paper, we present a systematic literature review investigating the areas of education where chatbots have already been applied, explore the pedagogical roles of chatbots, the use of chatbots for mentoring purposes, and their potential to personalize education. We conducted a preliminary analysis of 2,678 publications to perform this literature review, which allowed us to identify 74 relevant publications for chatbots’ application in education. Through this, we address five research questions that, together, allow us to explore the current state-of-the-art of this educational technology. We conclude our systematic review by pointing to three main research challenges: 1) Aligning chatbot evaluations with implementation objectives, 2) Exploring the potential of chatbots for mentoring students, and 3) Exploring and leveraging adaptation capabilities of chatbots. For all three challenges, we discuss opportunities for future research.

## Introduction

Educational Technologies enable distance learning models and provide students with the opportunity to learn at their own pace. They have found their way into schools and higher education institutions through Learning Management Systems and Massive Open Online Courses, enabling teachers to scale up good teaching practices (Ferguson and Sharples, 2014) and allowing students to access learning material ubiquitously (Virtanen et al., 2018).

Despite the innovative power of educational technologies, most commonly used technologies do not substantially change teachers’ role. Typical teaching activities like providing students with feedback, motivating them, or adapting course content to specific student groups are still entrusted exclusively to teachers, even in digital learning environments. This can lead to the teacher-bandwidth problem (Wiley and Edwards, 2002), the result of a shortage of teaching staff to provide highly informative and competence-oriented feedback at large scale. Nowadays, however, computers and other digital devices open up far-reaching possibilities that have not yet been fully exploited. For example, incorporating process data can provide students with insights into their learning progress and bring new possibilities for formative feedback, self-reflection, and competence development (Quincey et al., 2019). According to (Hattie, 2009), feedback in terms of learning success has a mean effect size of d = 0.75, while (Wisniewski et al., 2019) even report a mean effect of d = 0.99 for highly informative feedback. Such feedback provides suitable conditions for self-directed learning (Winne and Hadwin, 2008) and effective metacognitive control of the learning process (Nelson and Narens, 1994).

One of the educational technologies designed to provide actionable feedback in this regard is Learning Analytics. Learning Analytics is defined as the research area that focuses on collecting traces that learners leave behind and using those traces to improve learning (Duval and Verbert, 2012; Greller and Drachsler, 2012). Learning Analytics can be used both by students to reflect on their own learning progress and by teachers to continuously assess the students’ efforts and provide actionable feedback. Another relevant educational technology is Intelligent Tutoring Systems. Intelligent Tutoring Systems are defined as computerized learning environments that incorporate computational models (Graesser et al., 2001) and provide feedback based on learning progress. Educational technologies specifically focused on feedback for help-seekers, comparable to raising hands in the classroom, are Dialogue Systems and Pedagogical Conversational Agents (Lester et al., 1997). These technologies can simulate conversational partners and provide feedback through natural language (McLoughlin and Oliver, 1998).

Research in this area has recently focused on chatbot technology, a subtype of dialog systems, as several technological platforms have matured and led to applications in various domains. Chatbots incorporate generic language models extracted from large parts of the Internet and enable feedback by limiting themselves to text or voice interfaces. For this reason, they have also been proposed and researched for a variety of applications in education (Winkler and Soellner, 2018). Recent literature reviews on chatbots in education (Winkler and Soellner, 2018; Hobert, 2019a; Hobert and Meyer von Wolff, 2019; Jung et al., 2020; Pérez et al., 2020; Smutny and Schreiberova, 2020; Pérez-Marín, 2021) have reported on such applications as well as design guidelines, evaluation possibilities, and effects of chatbots in education.

In this paper, we contribute to the state-of-the-art of chatbots in education by presenting a systematic literature review, where we examine so-far unexplored areas such as implementation objectives, pedagogical roles, mentoring scenarios, the adaptations of chatbots to learners, and application domains. This paper is structured as follows: First, we review related work (section 2), derive research questions from it, then explain the applied method for searching related studies (section 3), followed by the results (section 4), and finally, we discuss the findings (section 5) and point to future research directions in the field (section 5).

## Related Work

In order to accurately cover the field of research and deal with the plethora of terms for chatbots in the literature (e.g. chatbot, dialogue system or pedagogical conversational agent) we propose the following definition:

Chatbots are digital systems that can be interacted with entirely through natural language via text or voice interfaces. They are intended to automate conversations by simulating a human conversation partner and can be integrated into software, such as online platforms, digital assistants, or be interfaced through messaging services.

Outside of education, typical applications of chatbots are in customer service (Xu et al., 2017), counseling of hospital patients (Vaidyam et al., 2019), or information services in smart speakers (Ram et al., 2018). One central element of chatbots is the intent classification, also named the Natural Language Understanding (NLU) component, which is responsible for the sense-making of human input data. Looking at the current advances in chatbot software development, it seems that this technology’s goal is to pass the Turing Test (Saygin et al., 2000) one day, which could make chatbots effective educational tools. Therefore, we ask ourselves “*Are we there yet? - Will we soon have an autonomous chatbot for every learner?”*

**FIGURE 1:**
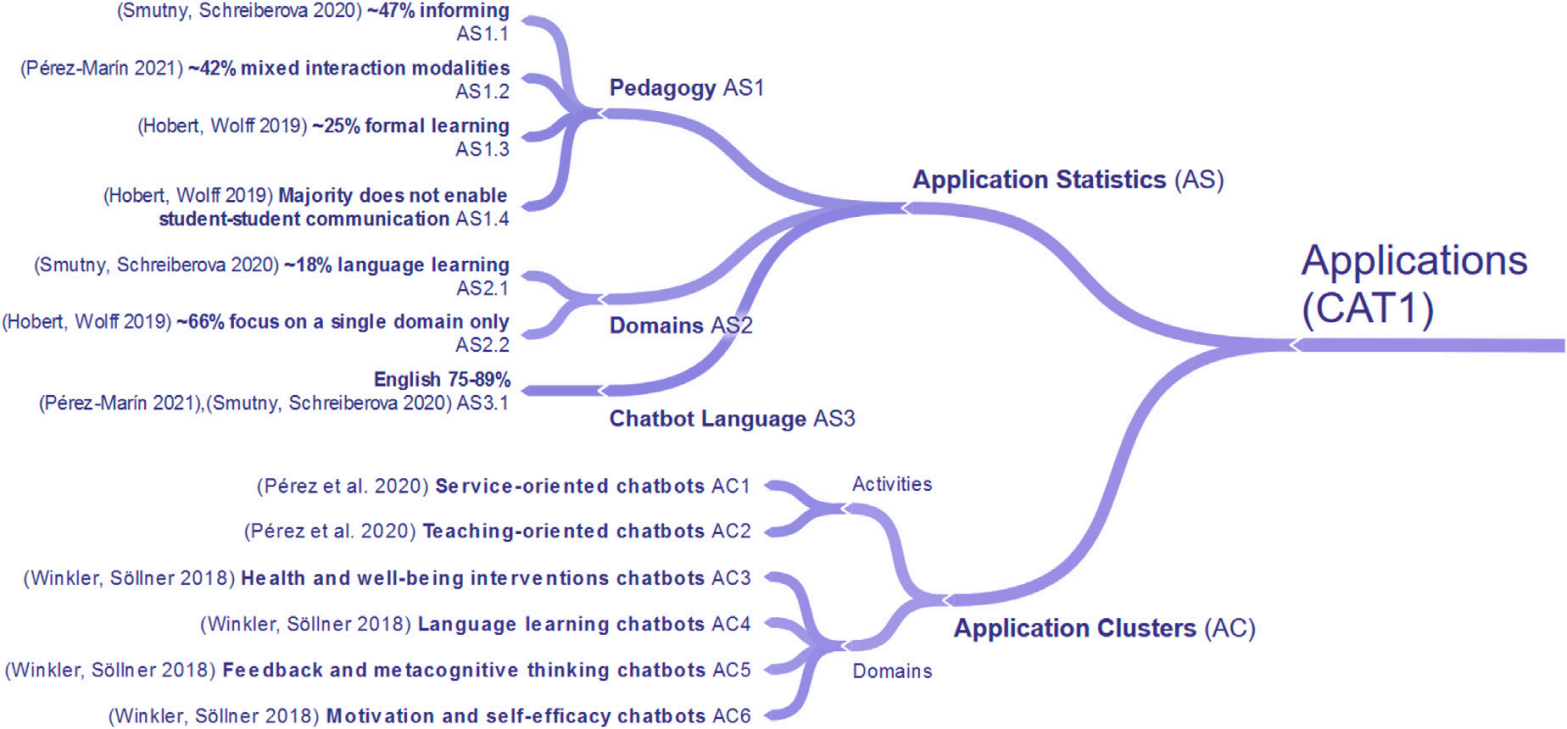
Applications of chatbots in related literature reviews (CAT1).

**FIGURE 2:**
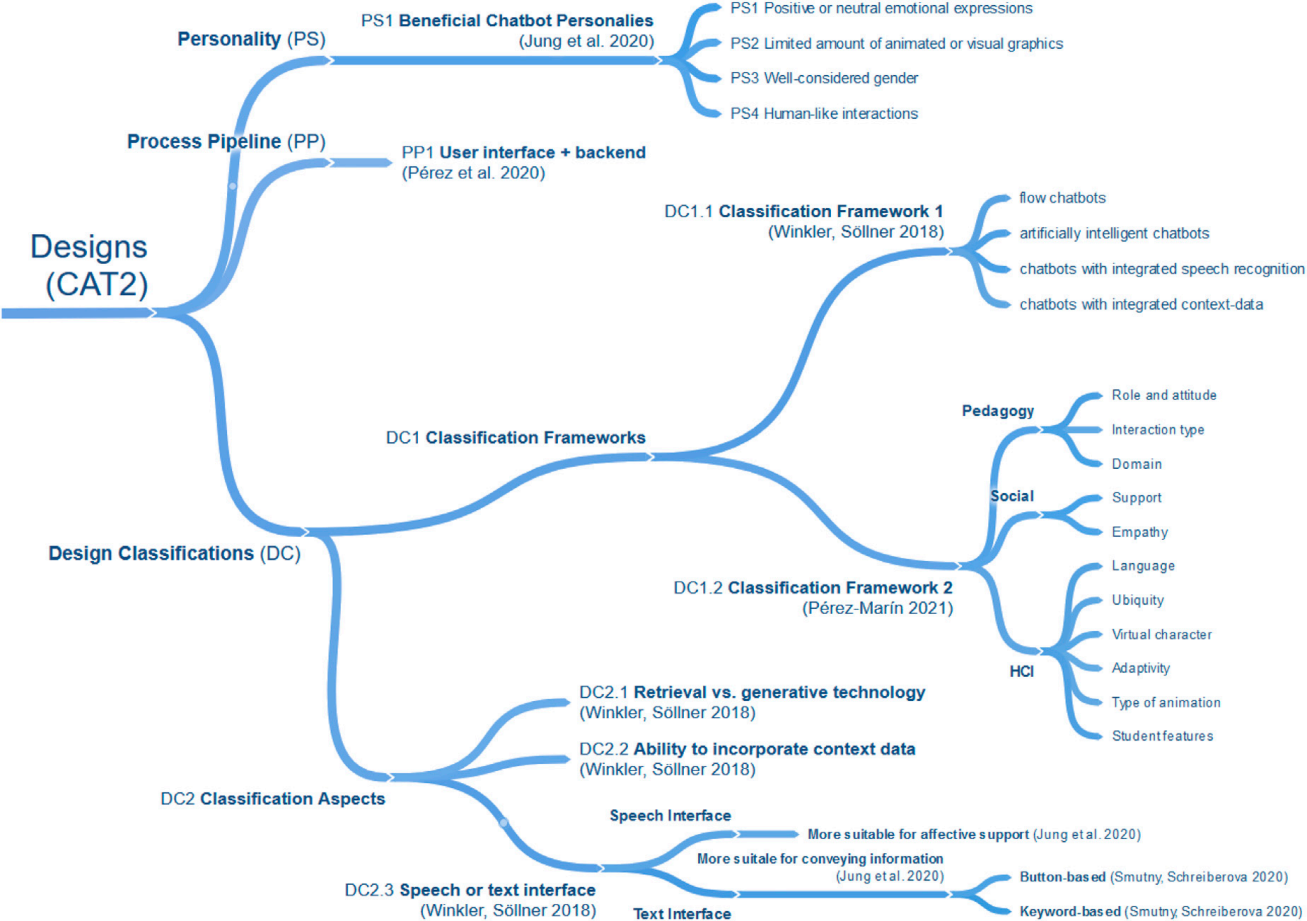
Chatbot designs in related literature reviews (CAT2).

**FIGURE 3:**
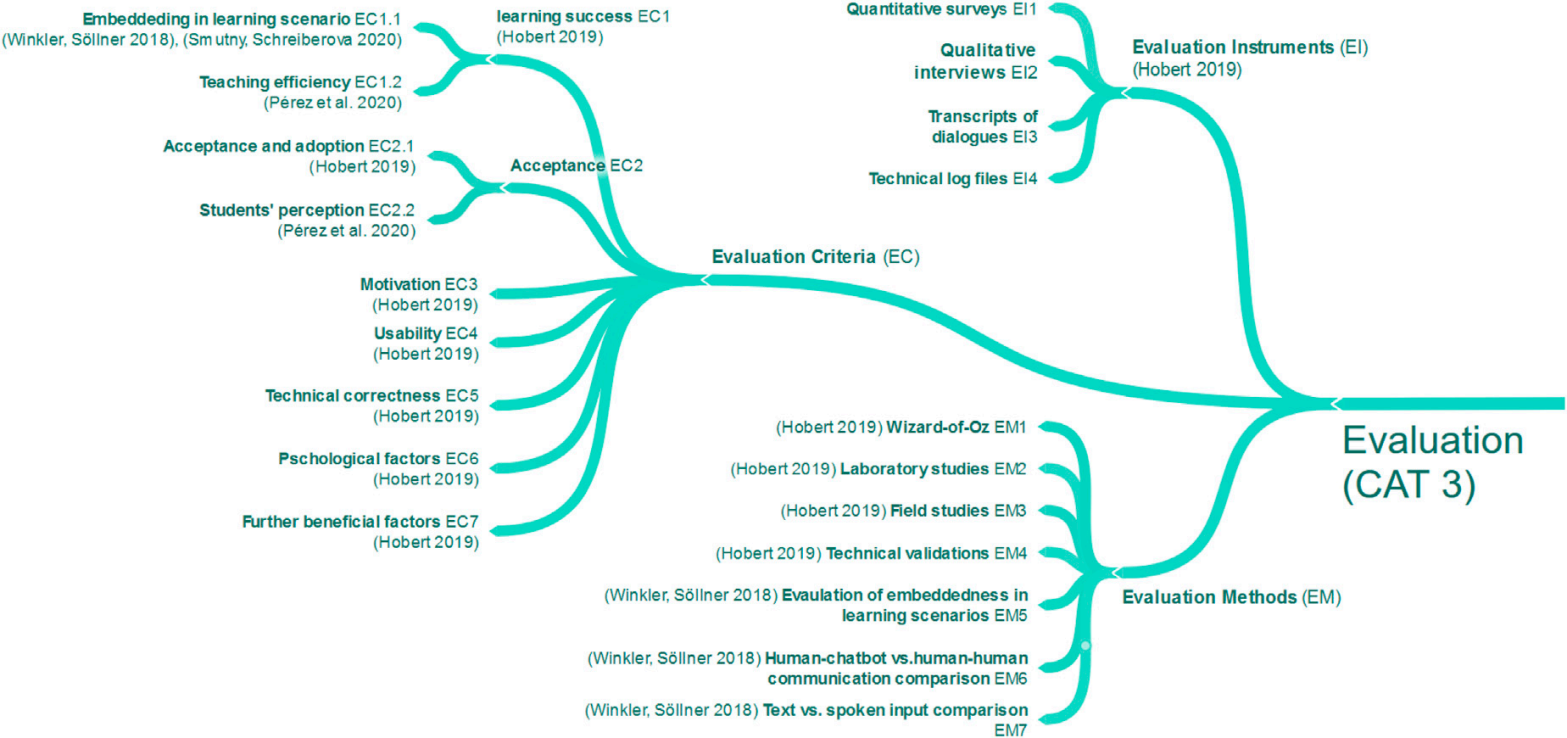
Evaluation of chatbots in related literature reviews (CAT3).

**FIGURE 4:**
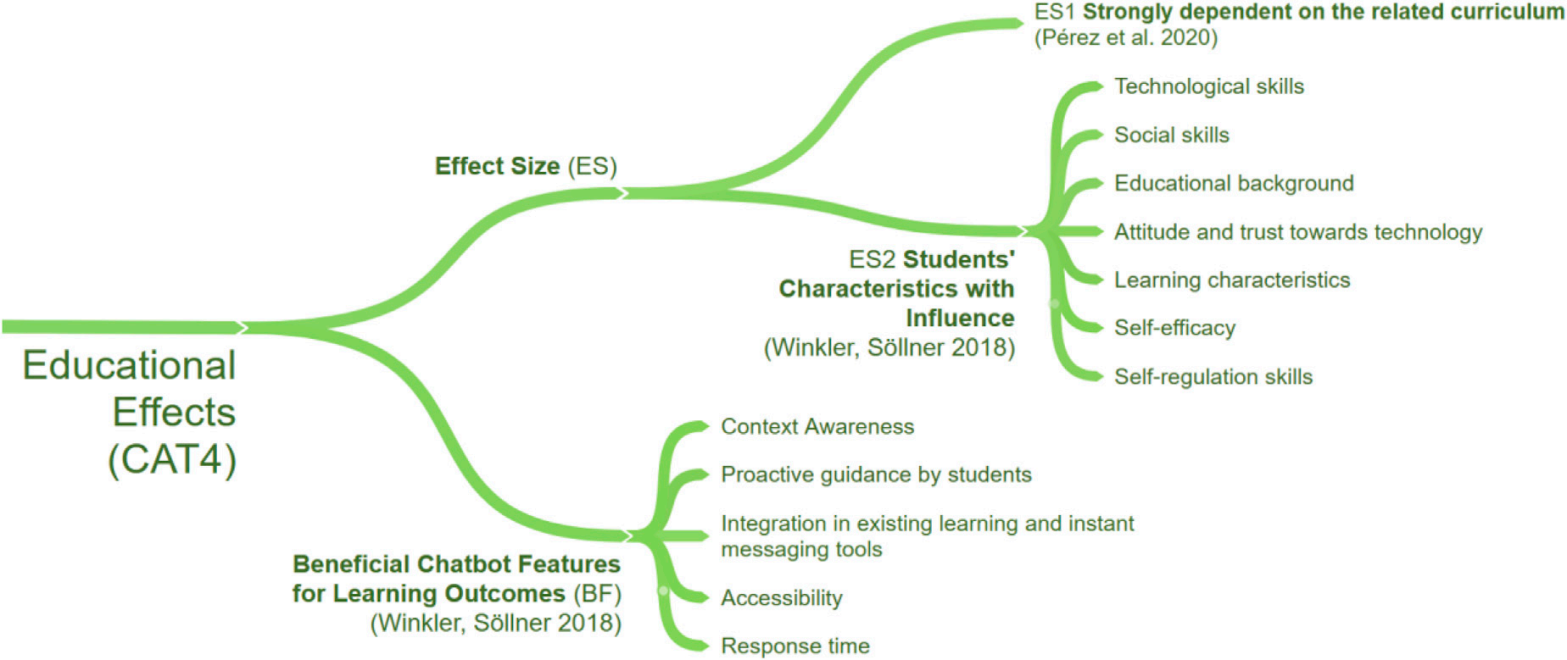
Educational Effects of chatbots in related literature reviews (CAT4).

To understand and underline the current need for research in the use of chatbots in education, we first examined the existing literature, focusing on comprehensive literature reviews. By looking at research questions in these literature reviews, we identified 21 different research topics and extracted findings accordingly. To structure research topics and findings in a comprehensible way, a three-stage clustering process was applied. While the first stage consisted of coding research topics by keywords, the second stage was applied to form overarching research categories (Table 1). In the final stage, the findings within each research category were clustered to identify and structure commonalities within the literature reviews. The result is a concept map, which consists of four major categories. Those categories are CAT1. Applications of Chatbots, CAT2. Chatbot Designs, CAT3. Evaluation of Chatbots and CAT4. Educational Effects of Chatbots. To standardize the terminology and concepts applied, we present the findings of each category in a separate sub-section, respectively (*see* Figure 1, Figure 2, Figure 3, and Figure 4) and extended it with the outcomes of our own literature study that will be reported in the remaining parts of this article. Due to the size of the concept map a full version can be found in **Appendix A**.

**TABLE 1:** Assignment of coded research topics identified in related literature reviews to research categories.

	CAT1: Applications	CAT2: Designs	CAT3: Evaluation	CAT4: Educational Effect
Pérez et al. (2020)	Application Clusters (AC)	Process Pipeline (PP)	Evaluation Criteria (EC)	Effect Size (ES)
Winkler and Soellner (2018)	Application Clusters (AC)	Design Classifications (DC)	Evaluation Criteria (EC), Evaluation Methods (EM)	Effect Size (ES), Beneficial Features (BF)
Smutny and Schreiberova (2020)	Application Statistics (AS)	Design Classifications (DC)	Evaluation Criteria (EC)	-
Jung et al. (2020)	-	Design Classifications (DC)	-	-
		Personality (PS)		
Hobert and Meyer von Wolff (2019)	Application Statistics (AS)	-	-	-
Pérez-Marín (2021)	Application Statistics (AS)	Design Classifications (DC)	-	-
Hobert (2019a)	-	-	Evaluation Criteria (EC)	-
			Evaluation Methods (EM)	
			Evaluation Instruments (EI)	

Regarding the applications of chatbots (CAT1), application clusters (AC) and application statistics (AS) have been described in the literature, which we visualized in Figure 1. The study of (Pérez et al., 2020) identifies two application clusters, defined through chatbot activities: “service-oriented chatbots” and “teaching-oriented chatbots.” (Winkler and Soellner, 2018) identify applications clusters by naming the domains “health and well-being interventions,” “language learning,” “feedback and metacognitive thinking” as well as “motivation and self-efficacy.” Concerning application statistics (AS), (Smutny and Schreiberova, 2020) found that nearly 47% of the analyzed chatbots incorporate informing actions, and 18% support language learning by elaborating on chatbots integrated into the social media platform Facebook. Besides, the chatbots studied had a strong tendency to use English, at 89%. This high number aligns with results from (Pérez-Marín, 2021), where 75% of observed agents, as a related technology, were designed to interact in the English language. (Pérez-Marín, 2021) also shows that 42% of the analyzed chatbots had mixed interaction modalities. Finally, (Hobert and Meyer von Wolff, 2019) observed that only 25% of examined chatbots were incorporated in formal learning settings, the majority of published material focuses on student-chatbot interaction only and does not enable student-student communication, as well as nearly two-thirds of the analyzed chatbots center only on a single domain. Overall, we can summarize that so far there are six application clusters for chatbots for education categorized by chatbot activities or domains. The provided statistics allow for a clearer understanding regarding the prevalence of chatbots applications in education (*see* Figure 1).

Regarding chatbot designs (CAT2), most of the research questions concerned with chatbots in education can be assigned to this category. We found three aspects in this category visualized in Figure 2: Personality (PS), Process Pipeline (PP), and Design Classifications (DC). Within these, most research questions can be assigned to Design Classifications (DC), which are separated into Classification Aspects (DC2) and Classification Frameworks (DC1). One classification framework is defined through “flow chatbots,” “artificially intelligent chatbots,” “chatbots with integrated speech recognition,” as well as “chatbots with integrated context-data” by (Winkler and Soellner, 2018). A second classification framework by (Pérez-Marín, 2021) covers pedagogy, social, and HCI features of chatbots and agents, which themselves can be further subdivided into more detailed aspects. Other Classification Aspects (DC2) derived from several publications, provide another classification schema, which distinguishes between “retrieval vs. generative” based technology, the “ability to incorporate context data,” and “speech or text interface” (Winkler and Soellner, 2018; Smutny and Schreiberova, 2020). By specifying text interfaces as “Button-Based” or “Keyword Recognition-Based” (Smutny and Schreiberova, 2020), text interfaces can be subdivided. Furthermore, a comparison of speech and text interfaces (Jung et al., 2020) shows that text interfaces have advantages for conveying information, and speech interfaces have advantages for affective support. The second aspect of CAT2 concerns the chatbot processing pipeline (PP), highlighting user interface and back-end importance (Pérez et al., 2020). Finally, (Jung et al., 2020) focuses on the third aspect, the personality of chatbots (PS). Here, the study derives four guidelines helpful in education: positive or neutral emotional expressions, a limited amount of animated or visual graphics, a well-considered gender of the chatbot, and human-like interactions. In summary, we have found in CAT2 three main design aspects for the development of chatbots. CAT2 is much more diverse than CAT1 with various sub-categories for the design of chatbots. This indicates the huge flexibility to design chatbots in various ways to support education.

Regarding the evaluation of chatbots (CAT3), we found three aspects assigned to this category, visualized in Figure 3: Evaluation Criteria (EC), Evaluation Methods (EM), and Evaluation Instruments (EI). Concerning Evaluation Criteria, seven criteria can be identified in the literature. The first and most important in the educational field, according to (Smutny and Schreiberova, 2020) is the evaluation of learning success (Hobert, 2019a), which can have subcategories such as how chatbots are embedded in learning scenarios (Winkler and Soellner, 2018; Smutny and Schreiberova, 2020) and teaching efficiency (Pérez et al., 2020). The second is acceptance, which (Hobert, 2019a) names as “acceptance and adoption” and (Pérez et al., 2020) as “students’ perception.” Further evaluation criteria are motivation, usability, technical correctness, psychological, and further beneficial factors (Hobert, 2019a). These Evaluation Criteria show broad possibilities for the evaluation of chatbots in education. However, (Hobert, 2019a) found that most evaluations are limited to single evaluation criteria or narrower aspects of them. Moreover, (Hobert, 2019a) introduces a classification matrix for chatbot evaluations, which consists of the following Evaluation Methods (EM): Wizard-of-Oz approach, laboratory studies, field studies, and technical validations. In addition to this, (Winkler and Soellner, 2018) recommends evaluating chatbots by their embeddedness into a learning scenario, a comparison of human-human and human-chatbot interactions, and comparing spoken and written communication. Instruments to measure these evaluation criteria were identified by (Hobert, 2019a) by naming quantitative surveys, qualitative interviews, transcripts of dialogues, and technical log files. Regarding CAT3, we found three main aspects for the evaluation of chatbots. We can conclude that this is a more balanced and structured distribution in comparison to CAT2, providing researchers with guidance for evaluating chatbots in education.

Regarding educational effects of chatbots (CAT4), we found two aspects visualized in Figure 4: Effect Size (ES) and Beneficial Chatbot Features for Learning Success (BF). Concerning the effect size, (Pérez et al., 2020) identified a strong dependency between learning and the related curriculum, while (Winkler and Soellner, 2018) elaborate on general student characteristics that influence how students interact with chatbots. They state that students’ attitudes towards technology, learning characteristics, educational background, self-efficacy, and self-regulation skills affect these interactions. Moreover, the study emphasizes chatbot features, which can be regarded as beneficial in terms of learning outcomes (BF): “Context-Awareness,” “Proactive guidance by students,” “Integration in existing learning and instant messaging tools,” “Accessibility,” and “Response Time.” Overall, for CAT4, we found two main distinguishing aspects for chatbots, however, the reported studies vary widely in their research design, making high-level results hardly comparable.

Looking at the related work, many research questions for the application of chatbots in education remain. Therefore, we selected five goals to be further investigated in our literature review. Firstly, we were interested in the objectives for implementing chatbots in education (Goal 1), as the relevance of chatbots for applications within education seems to be not clearly delineated. Secondly, we aim to explore the pedagogical roles of chatbots in the existing literature (Goal 2) to understand how chatbots can take over tasks from teachers. (Winkler and Soellner, 2018) and (Pérez-Marín, 2021), identified research gaps for supporting meta-cognitive skills with chatbots such as self-regulation. This requires a chatbot application that takes a mentoring role, as the development of these meta-cognitive skills can not be achieved solely by information delivery. Within our review we incorporate this by reviewing the mentoring role of chatbots as (Goal 3). Another key element for a mentoring chatbot is adaptation to the learners needs. Therefore, (Goal 4) of our review lies in the investigation of the adaptation approaches used by chatbots in education. For (Goal 5), we want to extend the work of (Winkler and Soellner, 2018) and (Pérez et al., 2020) regarding Application Clusters (AC) and map applications by further investigating specific learning domains in which chatbots have been studied.

## Methods

To delineate and map the field of chatbots in education, initial findings were collected by a preliminary literature search. One of the takeaways is that the emerging field around educational chatbots has seen much activity in the last two years. Based on the experience of this preliminary search, search terms, queries, and filters were constructed for the actual structured literature review. This structured literature review follows the PRISMA framework (Liberati et al., 2009), a guideline for reporting systematic reviews and meta-analyses. The framework consists of an elaborated structure for systematic literature reviews and sets requirements for reporting information about the review process (*see* section 3.2 to 3.4).

### Research Questions

Contributing to the state-of-the-art, we investigate five aspects of chatbot applications published in the literature. We therefore guided our research with the following research questions:

**RQ1:** Which objectives for implementing chatbots in education can be identified in the existing literature?

**RQ2:** Which pedagogical roles of chatbots can be identified in the existing literature?

**RQ3:** Which application scenarios have been used to mentor students?

**RQ4:** To what extent are chatbots adaptable to personal students’ needs?

**RQ5:** What are the domains in which chatbots have been applied so far?

### Sources of Information

As data sources, Scopus, Web of Science, Google Scholar, Microsoft Academics, and the educational research database “Fachportal Pädagogik” (including ERIC) were selected, all of which incorporate all major publishers and journals. In (Martín-Martín et al., 2018) it was shown that for the social sciences only 29.8% and for engineering and computer science, 46.8% of relevant literature is included in all of the first three databases. For the topic of chatbots in education, a value between these two numbers can be assumed, which is why an approach of integrating several publisher-independent databases was employed here.

### Search Criteria

Based on the findings from the initial related work search, we derived the following search query:

(*Education* OR *Educational* OR *Learning* OR *Learner* OR *Student* OR *Teaching* OR *School* OR *University* OR *Pedagogical*) AND Chatbot.

It combines education-related keywords with the “chatbot” keyword. Since chatbots are related to other technologies, the initial literature search also considered keywords such as “pedagogical agents,” “dialogue systems,” or “bots” when composing the search query. However, these increased the number of irrelevant results significantly and were therefore excluded from the query in later searches.

### Inclusion and Exclusion Criteria

**FIGURE 5:**
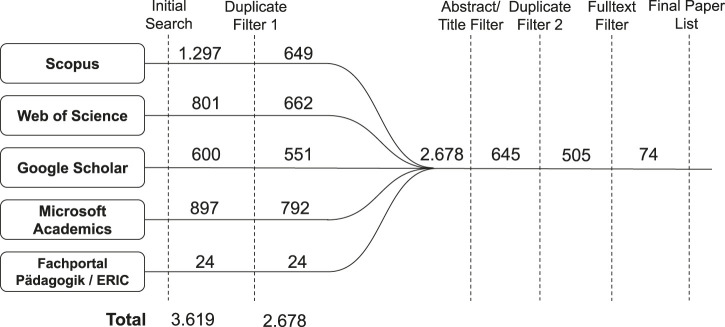
PRISMA flow chart.

The queries were executed on 23.12.2020 and applied twice to each database, first as a title search query and secondly as a keyword-based search. This resulted in a total of 3.619 hits, which were checked for duplicates resulting in 2.678 candidate publications. The overall search and filtering process is shown in Figure 5.

In the case of Google Scholar, the number of results sorted by relevance per query was limited to 300, as this database also delivers many less relevant works. The value was determined by looking at the search results in detail using several queries to exclude as few relevant works as possible. This approach showed promising results and, at the same time, did not burden the literature list with irrelevant items.

The further screening consisted of a four-stage filtering process. First, eliminating duplicates in the results of title and keyword queries of all databases independently and second, excluding publications based on the title and abstract that:

• were not available in English
• did not describe a chatbot application
• were not mainly focused on learner-centered chatbots applications in schools or higher education institutions, which is according to the preliminary literature search the main application area within education.

Third, we applied another duplicate filter, this time for the merged set of publications. Finally, a filter based on the full text, excluding publications that were:

• limited to improve chatbots technically (e.g., publications that compare or develop new algorithms), as research questions presented in these publications were not seeking for additional insights on applications in education
• exclusively theoretical in nature (e.g., publications that discuss new research projects, implementation concepts, or potential use cases of chatbots in education), as they either do not contain research questions or hypotheses or do not provide conclusions from studies with learners.

After the first, second, and third filters, we identified 505 candidate publications. We continued our filtering process by reading the candidate publications’ full texts resulting in 74 publications that were used for our review. Compared to 3.619 initial database results, the proportion of relevant publications is therefore about 2.0%.

The final publication list can be accessed under https://bit.ly/2RRArFT.

### Analysis

To analyze the identified publications and derive results according to the research questions, full texts were coded, considering for each publication the objectives for implementing chatbots (RQ1), pedagogical roles of chatbots (RQ2), their mentoring roles (RQ3), adaptation of chatbots (RQ4), as well as their implementation domains in education (RQ5) as separated sets of codes. To this end, initial codes were identified by open coding and iteratively improved through comparison, group discussion among the authors, and subsequent code expansion. Further, codes were supplemented with detailed descriptions until a saturation point was reached, where all included studies could be successfully mapped to codes, suggesting no need for further refinement. As an example, codes for RQ2 (Pedagogical Roles) were adapted and refined in terms of their level of abstraction from an initial set of only two codes, *1*) a code for chatbots in the learning role and *2*) a code for chatbots in a service-oriented role. After coding a larger set of publications, it became clear that the code for service-oriented chatbots needed to be further distinguished. This was because it summarized e.g. automation activities with activities related to self-regulated learning and thus could not be distinguished sharply enough from the learning role. After refining the code set in the next iteration into a learning role, an assistance role, and a mentoring role, it was then possible to ensure the separation of the individual codes. In order to avoid defining new codes for singular or a very small number of publications, studies were coded as “other” (RQ1) or “not defined” (RQ2), if their occurrence was less than eight publications, representing less than 10% of the publications in the final paper list.

## Results

**FIGURE 6:**
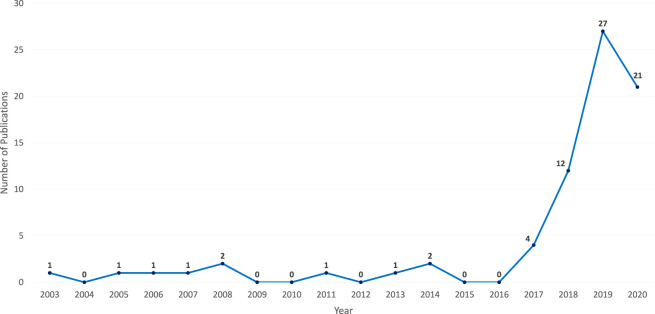
Identified chatbot publications in education per year.

By grouping the resulting relevant publications according to their date of publication, it is apparent that chatbots in education are currently in a phase of increased attention. The release distribution shows slightly lower publication numbers in the current than in the previous year (Figure 6), which could be attributed to a time lag between the actual publication of manuscripts and their dissemination in databases.

Applying the curve presented in Figure 6 to Gartner’s Hype Cycle (Linden and Fenn, 2003) suggests that technology around chatbots in education may currently be in the “Innovation Trigger” phase. This phase is where many expectations are placed on the technology, but the practical in-depth experience is still largely lacking.

### Objectives for Implementing Chatbots in Education

**FIGURE 7:**
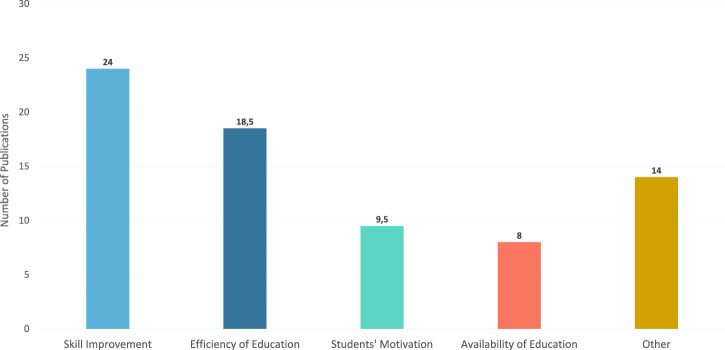
Objectives for implementing chatbots identified in chatbot publications.

Regarding RQ1, we extracted implementation objectives for chatbots in education. By analyzing the selected publications we identified that most of the objectives for chatbots in education can be described by one of the following categories: Skill improvement, Efficiency of Education, and Students’ Motivation (*see* Figure 7). First, the “improvement of a student’s skill” (or *Skill Improvement*) objective that the chatbot is supposed to help with or achieve. Here, chatbots are mostly seen as a learning aid that supports students. It is the most commonly cited objective for chatbots. The second objective is to increase the *Efficiency of Education* in general. It can occur, for example, through the automation of recurring tasks or time-saving services for students and is the second most cited objective for chatbots. The third objective is to increase *Students’ Motivation*. Finally, the last objective is to increase the *Availability of Education*. This objective is intended to provide learning or counseling with temporal flexibility or without the limitation of physical presence. In addition, there are other, more diverse objectives for chatbots in education that are less easy to categorize. In cases of a publication indicating more than one objective, the publication was distributed evenly across the respective categories.

Given these results, we can summarize four major implementing objectives for chatbots. Of these, *Skill Improvement* is the most popular objective, constituting around one-third of publications (32%). Making up a quarter of all publications, *Efficiency of Education* is the second most popular objective (25%), while addressing *Students’ Motivation* and *Availability of Education* are third (13%) and fourth (11%), respectively. *Other* objectives also make up a substantial amount of these publications (19%), although they were too diverse to categorize in a uniform way. Examples of these are inclusivity (Heo and Lee, 2019) or the promotion of student teacher interactions (Mendoza et al., 2020).

### Pedagogical Roles

Regarding RQ2, it is crucial to consider the use of chatbots in terms of their intended pedagogical role. After analyzing the selected articles, we were able to identify four different pedagogical roles: a supporting learning role, an assisting role, and a mentoring role.

In the supporting learning role (*Learning*), chatbots are used as an educational tool to teach content or skills. This can be achieved through a fixed integration into the curriculum, such as conversation tasks (L. K. Fryer et al., 2020). Alternatively, learning can be supported through additional offerings alongside classroom teaching, for example, voice assistants for leisure activities at home (Bao, 2019). Examples of these are chatbots simulating a virtual pen pal abroad (Na-Young, 2019). Conversations with this kind of chatbot aim to motivate the students to look up vocabulary, check their grammar, and gain confidence in the foreign language.

In the assisting role (*Assisting*), chatbot actions can be summarized as simplifying the student's everyday life, i.e., taking tasks off the student’s hands in whole or in part. This can be achieved by making information more easily available (Sugondo and Bahana, 2019) or by simplifying processes through the chatbot’s automation (Suwannatee and Suwanyangyuen, 2019). An example of this is the chatbot in (Sandoval, 2018) that answers general questions about a course, such as an exam date or office hours.

In the mentoring role (*Mentoring*), chatbot actions deal with the student’s personal development. In this type of support, the student himself is the focus of the conversation and should be encouraged to plan, reflect or assess his progress on a meta-cognitive level. One example is the chatbot in (Cabales, 2019), which helps students develop lifelong learning skills by prompting in-action reflections.

**FIGURE 8:**
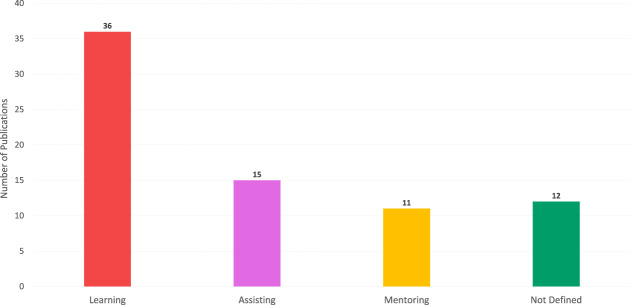
Pedagogical roles identified in chatbot publications.

The distribution of each pedagogical role is shown in Figure 8. From this, it can be seen that *Learning* is the most frequently used role of the examined publications (49%), followed by *Assisting* (20%) and *Mentoring* (15%). It should be noted that pedagogical roles were not identified for all the publications examined. The absence of a clearly defined pedagogical role (16%) can be attributed to the more general nature of these publications, e.g. focused on students’ small talk behaviors (Hobert, 2019b) or teachers’ attitudes towards chatbot applications in classroom teaching (P. K. Bii et al., 2018).

**FIGURE 9:**
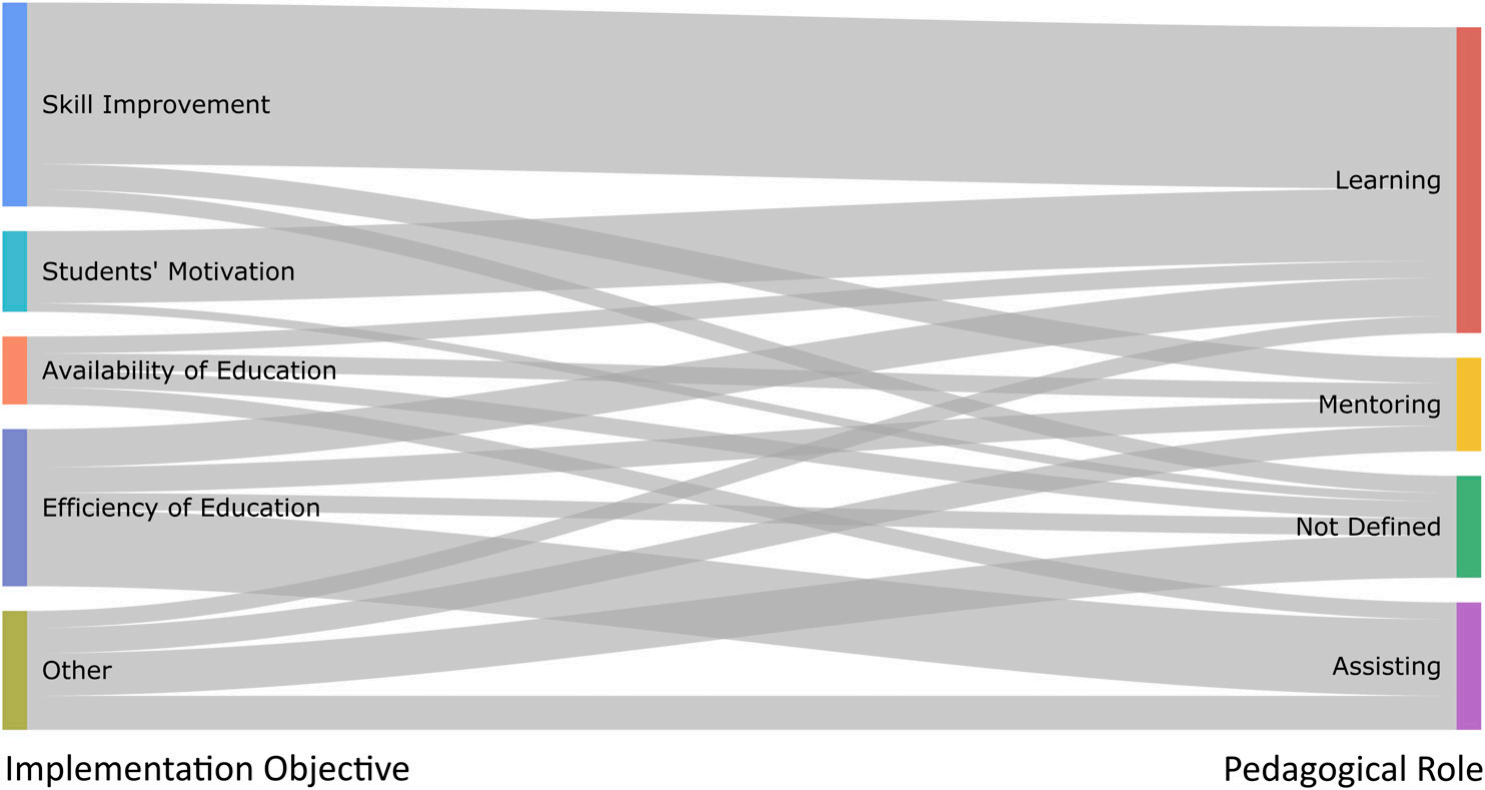
Relations graph of pedagogical roles and objectives for implementing chatbots.

Looking at pedagogical roles in the context of objectives for implementing chatbots, relations among publications can be inspected in a relations graph (Figure 9). According to our results, the strongest relation in the examined publications can be considered between *Skill Improvement* objective and the *Learning* role. This strong relation is partly because both, the *Skill Improvement* objective and the *Learning* role, are the largest in their respective categories. In addition, two other strong relations can be observed: Between the *Students’ Motivation* objective and the *Learning* role, as well as between *Efficiency of Education* objective and *Assisting* role.

By looking at other relations in more detail, there is surprisingly no relation between *Skill Improvement* as the most common implementation objective and *Assisting*, as the 2nd most common pedagogical role. Furthermore, it can be observed that the *Mentoring* role has nearly equal relations to all of the objectives for implementing chatbots.

The relations graph (Figure 9) can interactively be explored through bit.ly/32FSKQM.

### Mentoring Role

Regarding RQ3, we identified eleven publications that deal with chatbots in this regard. The *Mentoring* role in these publications can be categorized in two dimensions. Starting with the first dimension, the mentoring method, three methods can be observed:

• *Scaffolding* (*n* = 7)
• *Recommending* (*n* = 3)
• *Informing* (*n* = 1)

An example of *Scaffolding* can be seen in (Gabrielli et al., 2020), where the chatbot coaches students in life skills, while an example of *Recommending* can be seen in (Xiao et al., 2019), where the chatbot recommends new teammates. Finally, *Informing* can be seen in (Kerly et al., 2008), where the chatbot informs students about their personal Open Learner Model.

The second dimension is the addressed mentoring topic, where the following topics can be observed:

• *Self-Regulated Learning* (*n* = 5)
• *Life Skills* (*n* = 4)
• *Learning Skills* (*n* = 2)

While *Mentoring* chatbots to support *Self-Regulated Learning* are intended to encourage students to reflect on and plan their learning progress, *Mentoring* chatbots to support *Life Skills* address general student’s abilities such as self-confidence or managing emotions. Finally, *Mentoring* chatbots to support *Learning Skills*, in contrast to *Self-Regulated Learning*, address only particular aspects of the learning process, such as new learning strategies or helpful learning partners. An example for *Mentoring* chatbots supporting *Life Skill* is the Logo counseling chatbot, which promotes healthy self-esteem (Engel et al., 2020). CALMsystem is an example of a *Self-Regulated Learning* chatbot, which informs students about their data in an open learner model (Kerly et al., 2008). Finally, there is the *Learning Skills* topic. Here, the MCQ Bot is an example that is designed to introduce students to transformative learning (W. Huang et al., 2019).

### Adaptation

Regarding RQ4, we identified six publications in the final publication list that address the topic of adaptation. Within these publications, five adaptation approaches are described:

The first approach (A1) is proposed by (Kerly and Bull, 2006) and (Kerly et al., 2008), dealing with student discussions based on success and confidence during a quiz. The improvement of self-assessment is the primary focus of this approach. The second approach (A2) is presented in (Jia, 2008), where the personality of the chatbot is adapted to motivate students to talk to the chatbot and, in this case, learn a foreign language. The third approach (A3), as shown in the work of (Vijayakumar et al., 2019), is characterized by a chatbot that provides personalized formative feedback to learners based on their self-assessment, again in a quiz situation. Here, the focus is on Hattie and Timperley’s three guiding questions: “Where am I going?,” “How am I going?” and “Where to go next?” (Hattie and Timperley, 2007). In the fourth approach (A4), exemplified in (Ruan et al., 2019), the chatbot selects questions within a quiz. Here, the chatbot estimates the student’s ability and knowledge level based on the quiz progress and sets the next question accordingly. Finally, a similar approach (A5) is shown in (Davies et al., 2020). In contrast to (Ruan et al., 2019), this chatbot adapts the amount of question variation and takes psychological features into account which were measured by psychological tests before.

We examined these five approaches by organizing them according to their information sources and extracted learner information. The results can be seen in Table 2.

**TABLE 2:** Adaptation approaches of chatbots in education.

Adaptation Approach	Information Source	Extracted learner Information
Discussing Learning Quiz Progress (A1) Kerly and Bull (2006); Kerly et al. (2008)	Students’ self-assessment, quiz results	Confidence, knowledge level
Adapting Chatbot Personality (A2) Jia (2008)	Registration questionnaire, dialogue data	Students’ interest
Formative Quiz Feedback (A3) Vijayakumar et al. (2019)	Students’ self-assessment, quiz results	Confidence, knowledge level
Quiz Question Selection (A4) Ruan et al. (2019)	Quiz progress	Ability, knowledge level
Quiz Question Variation Adaptation (A5) Davies et al. (2020)	Psychological tests	Psychological features


Four out of five adaptation approaches (A1, A3, A4, and A5) are observed in the context of quizzes. These adaptations within quizzes can be divided into two mainstreams: One is concerned about students’ feedback (A1 and A3), while the other is concerned about learning material selection (A4 and A5). The only different adaptation approach is shown in A2, which focuses on the adaptation of the chatbot personality within a language learning application.

### Domains for Chatbots in Education

Regarding RQ5, we identified 20 domains of chatbots in education. These can broadly be divided by their pedagogical role into three domain categories (DC): *Learning Chatbots*, *Assisting Chatbots*, and *Mentoring Chatbots*. The remaining publications are grouped in the *Other Research* domain category. The complete list of identified domains can be seen in Table 3.

**TABLE 3:** Domains of chatbots in education.

Domain Category	Domain
Learning Chatbots (*n* = 36)	Language Learning (*n* = 19)
	Bao (2019); Chen et al. (2020); Davies et al. (2020); Fryer et al. (2017); Fryer et al. (2019); Fryer et al. (2020); Huang et al. (2018); Jia (2004), (2008); Haristiani; Nagata et al. (2020); Na-Young (2017), (2018c), (2018a), (2018b), (2019); Nghi et al. (2019); Pham et al. (2018); Goda et al. (2014)
	Learn to Program (*n* = 7)
	Abbasi and Kazi (2014); Daud et al. (2020); Ismail and Ade-Ibijola (2019); Kerly et al. (2007); Kerly and Bull (2006); Lin and Tsai (2019); Subramaniam (2019)
	Mathematics Learning (*n* = 2)
	(Febriani and Agustia, 2019; Yin et al., 2020)
	Learning Chatbot Frameworks (*n* = 2)
	Ruan et al. (2019); Vijayakumar et al. (2019)
	Learn Communication Skills (*n* = 1)
	Chang and Hwang (2019)
	Learn about Educational Technologies (*n* = 1)
	Liu et al. (2020)
	Learn about Laws (*n* = 1)
	Diachenko et al. (2019)
	Learn Writing Skills (*n* = 1)
	Lin and Chang (2020)
	Learn about Psychology (*n* = 1)
	Heller et al. (2005)
	Learn about Computer Administration (*n* = 1)
	Hsieh (2011)
Assisting Chatbots (*n* = 15)	Administrative Assistance (*n* = 5)
	Chen et al. (2018); Galko et al. (2018); Lee et al. (2019); Mamani et al. (2019); Rebaque-Rivas and Gil-Rodríguez (2019)
	Campus Assistance (*n* = 4)
	Heo an Lee (2019); Ondas et al. (2019); Meyer et al. (2020); Stapić et al. (2020)
	Course Assistance (*n* = 4)
	Carayannopoulos (2018); Mendoza et al. (2020); Sandoval (2018); Sugondo and Bahana (2019)
	Library Assistance (*n* = 2)
	Kumar et al. (2016); Suwannatee and Suwanyangyuen (2019)
Mentoring Chatbots (*n* = 11)	Scaffolding Chatbots (*n* = 7)
	Cabales (2019); Durall and Kapros (2020); Engel et al. (2020); Gabrielli et al. (2020); Huang et al. (2019); Matsuura and Omokawa (2020); Wolfbauer et al. (2020)
	Recommending Chatbots (*n* = 3)
	Chan et al. (2018); Robinson (2019); Xiao et al. (2019)
	Informing Chatbots (*n* = 1)
	Kerly et al. (2008)
Other Research (*n* = 12)	General Chatbot Research in Education (*n* = 7)
	Abbasi et al. (2019); Almahri et al. (2020); Bii et al. (2018); Bos et al. (2020); Hobert (2020); Pereira et al. (2019); Song et al. (2019)
	Chatbot Interfaces (*n* = 3)
	Matsuura and Ishimura (2017); Dibitonto et al. (2018); Sinclair et al. (2019)
	Indian Educational System (*n* = 2)
	(Bii et al. (2013); Sandu and Gide (2019)

The domain category *Learning Chatbots*, which deals with chatbots incorporating the pedagogical role *Learning*, can be subdivided into seven domains: *1*) *Language Learning*, *2*) *Learn to Program*, *3*) *Learn Communication Skills*, *4*) *Learn about Educational Technologies*, *5*) *Learn about Cultural Heritage*, *6*) *Learn about Laws*, and *7*) *Mathematics Learning*. With more than half of publications (53%), chatbots for *Language Learning* play a prominent role in this domain category. They are often used as chat partners to train conversations or to test vocabulary. An example of this can be seen in the work of (Bao, 2019), which tries to mitigate foreign language anxiety by chatbot interactions in foreign languages.

The domain category *Assisting Chatbots*, which deals with chatbots incorporating the pedagogical role *Assisting*, can be subdivided into four domains: *1*) *Administrative Assistance*, *2*) *Campus Assistance*, *3*) *Course Assistance*, and *4*) *Library Assistance*. With one-third of publications (33%), chatbots in the *Administrative Assistance* domain that help to overcome bureaucratic hurdles at the institution, while providing round-the-clock services, are the largest group in this domain category. An example of this can be seen in (Galko et al., 2018), where the student enrollment process is completely shifted to a conversation with a chatbot.

The domain category *Mentoring Chatbots*, which deals with chatbots incorporating the pedagogical role *Mentoring*, can be subdivided into three domains: *1*) *Scaffolding Chatbots*, *2*) *Recommending Chatbots*, and *3*) *Informing Chatbots*. An example of a *Scaffolding Chatbots* is the CRI(S) chatbot (Gabrielli et al., 2020), which supports life skills such as self-awareness or conflict resolution in discussion with the student by promoting helpful ideas and tricks.

The domain category *Other Research*, which deals with chatbots not incorporating any of these pedagogical roles, can be subdivided into three domains: *1*) *General Chatbot Research in Education*, *2*) *Indian Educational System*, and *3*) *Chatbot Interfaces*. The most prominent domain, *General Chatbot Research*, cannot be classified in one of the other categories but aims to explore cross-cutting issues. An example for this can be seen in the publication of (Hobert, 2020), which researches the importance of small talk abilities of chatbots in educational settings.

## Discussions

In this paper, we investigated the state-of-the-art of chatbots in education according to five research questions. By combining our results with previously identified findings from related literature reviews, we proposed a concept map of chatbots in education. The map, reported in **Appendix A**, displays the current state of research regarding chatbots in education with the aim of supporting future research in the field.

### Answer to Research Questions

Concerning RQ1 (implementation objectives), we identified four major objectives: *1*) *Skill Improvement*, *2*) *Efficiency of Education*, *3*) *Students’ Motivation,* and *4*) *Availability of Education*. These four objectives cover over 80% of the analyzed publications (*see* Figure 7). Based on the findings on CAT3 in section 2, we *see* a mismatch between the objectives for implementing chatbots compared to their evaluation. Most researchers only focus on narrow aspects for the evaluation of their chatbots such as learning success, usability, and technology acceptance. This mismatch of implementation objectives and suitable evaluation approaches is also well known by other educational technologies such as Learning Analytics dashboards (Jivet et al., 2017). A more structured approach of aligning implementation objectives and evaluation procedures is crucial to be able to properly assess the effectiveness of chatbots. (Hobert, 2019a), suggested a structured four-stage evaluation procedure beginning with a Wizard-of-Oz experiment, followed by technical validation, a laboratory study, and a field study. This evaluation procedure systematically links hypotheses with outcomes of chatbots helping to assess chatbots for their implementation objectives. “Aligning chatbot evaluations with implementation objectives” is, therefore, an important challenge to be addressed in the future research agenda.

Concerning RQ2 (pedagogical roles), our results show that chatbots’ pedagogical roles can be summarized as *Learning*, *Assisting*, and *Mentoring*. The *Learning* role is the support in learning or teaching activities such as gaining knowledge. The *Assisting* role is the support in terms of simplifying learners’ everyday life, e.g. by providing opening times of the library. The *Mentoring* role is the support in terms of students’ personal development, e.g. by supporting Self-Regulated Learning. From a pedagogical standpoint, all three roles are essential for learners and should therefore be incorporated in chatbots. These pedagogical roles are well aligned with the four implementation objectives reported in RQ1. While *Skill Improvement* and *Students’ Motivation* is strongly related to *Learning*, *Efficiency of Education* is strongly related to *Assisting*. The *Mentoring* role instead, is evenly related to all of the identified objectives for implementing chatbots. In the reviewed publications, chatbots are therefore primarily intended to *1*) improve skills and motivate students by supporting learning and teaching activities, *2*) make education more efficient by providing relevant administrative and logistical information to learners, and *3*) support multiple effects by mentoring students.

Concerning RQ3 (mentoring role), we identified three main mentoring method categories for chatbots: *1*) *Scaffolding*, *2*) *Recommending*, and *3*) *Informing*. However, comparing the current mentoring of chatbots reported in the literature with the daily mentoring role of teachers, we can summarize that the chatbots are not at the same level. In order to take over mentoring roles of teachers (Wildman et al., 1992), a chatbot would need to fulfill some of the following activities in their mentoring role. With respect to *1*) *Scaffolding*, chatbots should provide direct assistance while learning new skills and especially direct beginners in their activities. Regarding *2*) *Recommending*, chatbots should provide supportive information, tools or other materials for specific learning tasks to life situations. With respect to *3*) *Informing,* chatbots should encourage students according to their goals and achievements, and support them to develop meta-cognitive skills like self-regulation. Due to the mismatch of teacher vs. chatbot mentoring we *see* here another research challenge, which we call “Exploring the potential of chatbots for mentoring students.”

Regarding RQ4 (adaptation), only six publications were identified that discuss an adaptation of chatbots, while four out of five adaptation approaches (A1, A3, A4, and A5) show similarities by being applied within quizzes. In the context of educational technologies, providing reasonable adaptations for learners requires a high level of experience. Based on our results, the research on chatbots does not seem to be at this point yet. Looking at adaptation literature like (Brusilovsky, 2001) or (Benyon and Murray, 1993), it becomes clear that a chatbot needs to consider the learners’ personal information to fulfill the requirement of the adaptation definition. Personal information must be retrieved and stored at least temporarily, in some sort of learner model. For learner information like knowledge and interest, adaptations seem to be barely explored in the reviewed publications, while the model of (Brusilovsky and Millán, 2007) points out further learner information, which can be used to make chatbots more adaptive: personal goals, personal tasks, personal background, individual traits, and the learner’s context. We identify research in this area as a third future challenge and call it the “Exploring and leveraging adaptation capabilities of chatbots” challenge.

In terms of RQ5 (domains), we identified a detailed map of domains applying chatbots in education and their distribution (*see* Table 3). By systematically analyzing 74 publications, we identified 20 domains and structured them according to the identified pedagogical role into four domain categories: *Learning Chatbots*, *Assisting Chatbots*, *Mentoring Chatbots*, and *Other Research*. These results extend the taxonomy of Application Clusters (AC) for chatbots in education, which previously comprised the work from (Pérez et al., 2020), who took the chatbot activity as characteristic, and (Winkler and Soellner, 2018), who characterized the chatbots by domains. It draws relationships between these two types of Application Clusters (AC) and structures them accordingly. Our structure incorporates *Mentoring Chatbots* and *Other Research* in addition to the “service-oriented chatbots” (cf. *Assisting Chatbots*) and “teaching-oriented chatbots” (cf. *Learning Chatbots*) identified by (Perez). Furthermore, the strong tendencies of informing students already mentioned by (Smutny and Schreiberova, 2020) can also be recognized in our results, especially in *Assisting Chatbots*. Compared to (Winkler and Soellner, 2018), we can confirm the prominent domains of “language learning” within *Learning Chatbots* and “metacognitive thinking” within *Mentoring Chatbots*. Moreover, through Table 3, a more detailed picture of chatbot applications in education is reflected, which could help researchers to find similar works or unexplored application areas.

### Limitations

One important limitation to be mentioned here is the exclusion of alternative keywords for our search queries, as we exclusively used chatbot as keyword in order to avoid search results that do not fit our research questions. Though we acknowledge that chatbots share properties with pedagogical agents, dialog systems, and bots, we carefully considered this trade-off between missing potentially relevant work and inflating our search procedure by including related but not necessarily pertinent work. A second limitation may lie in the formation of categories and coding processes applied, which, due to the novelty of the findings, could not be built upon theoretical frameworks or already existing code books. Although we have focused on ensuring that codes used contribute to a strong understanding, the determination of the abstraction level might have affected the level of detail of the resulting data representation.

## Conclusion

In this systematic literature review, we explored the current landscape of chatbots in education. We analyzed 74 publications, identified 20 domains of chatbots and grouped them based on their pedagogical roles into four domain categories. These pedagogical roles are the supporting learning role (*Learning*), the assisting role (*Assisting*), and the mentoring role (*Mentoring*). By focusing on objectives for implementing chatbots, we identified four main objectives: *1*) *Skill Improvement*, *2*) *Efficiency of Education*, *3*) *Students’ Motivation,* and *4*) *Availability of Education*. As discussed in section 5, these objectives do not fully align with the chosen evaluation procedures. We focused on the relations between pedagogical roles and objectives for implementing chatbots and identified three main relations: *1*) chatbots to improve skills and motivate students by supporting learning and teaching activities, *2*) chatbots to make education more efficient by providing relevant administrative and logistical information to learners, and *3*) chatbots to support multiple effects by mentoring students. We focused on chatbots incorporating the *Mentoring* role and found that these chatbots are mostly concerned with three mentoring topics *1*) *Self-Regulated Learning*, *2*) *Life Skills*, and *3*) *Learning Skills* and three mentoring methods *1*) *Scaffolding*, *2*) *Recommending*, and *3*) *Informing*. Regarding chatbot adaptations, only six publications with adaptations were identified. Furthermore, the adaptation approaches found were mostly limited to applications within quizzes and thus represent a research gap.

Based on these outcomes we consider three challenges for chatbots in education that offer future research opportunities:

Challenge 1: *Aligning chatbot evaluations with implementation objectives*. Most chatbot evaluations focus on narrow aspects to measure the tool’s usability, acceptance or technical correctness. If chatbots should be considered as learning aids, student mentors, or facilitators, the effects on the cognitive, and emotional levels should also be taken into account for the evaluation of chatbots. This finding strengthens our conclusion that chatbot development in education is still driven by technology, rather than having a clear pedagogical focus of improving and supporting learning.

Challenge 2: *Exploring the potential of chatbots for mentoring students*. In order to better understand the potentials of chatbots to mentor students, more empirical studies on the information needs of learners are required. It is obvious that these needs differ from schools to higher education. However, so far there are hardly any studies investigating the information needs with respect to chatbots nor if chatbots address these needs sufficiently.

Challenge 3: *Exploring and leveraging adaptation capabilities of chatbots*. There is a large literature on adaptation capabilities of educational technologies. However, we have seen very few studies on the effect of adaptation of chatbots for education purposes. As chatbots are foreseen as systems that should personally support learners, the area of adaptable interactions of chatbots is an important research aspect that should receive more attention in the near future.

By addressing these challenges, we believe that chatbots can become effective educational tools capable of supporting learners with informative feedback. Therefore, looking at our results and the challenges presented, we conclude, “No, we are not there yet!” - There is still much to be done in terms of research on chatbots in education. Still, development in this area seems to have just begun to gain momentum and we expect to *see* new insights in the coming years.

## Data Availability

The original contributions presented in the study are included in the article/supplementary material, further inquiries can be directed to the corresponding authors.

## References

[B1] Abbasi S. Kazi H. Hussaini N. N. (2019). Effect of Chatbot Systems on Student’s Learning Outcomes. Sylwan 163(10).

[B2] Abbasi S. Kazi H. (2014). Measuring Effectiveness of Learning Chatbot Systems on Student's Learning Outcome and Memory Retention. Asian J. Appl. Sci. Eng. 3, 57. 10.15590/AJASE/2014/V3I7/53576

[B3] Almahri F. A. J. Bell D. Merhi M. (2020). “Understanding Student Acceptance and Use of Chatbots in the United Kingdom Universities: A Structural Equation Modelling Approach,” in 2020 6th IEEE International Conference on Information Management, ICIM 2020, London, United Kingdom, March 27–29, 2020, (IEEE), 284–288. 10.1109/ICIM49319.2020.244712

[B4] Bao M. (2019). Can Home Use of Speech-Enabled Artificial Intelligence Mitigate Foreign Language Anxiety - Investigation of a Concept. Awej 5, 28–40. 10.24093/awej/call5.3

[B5] Benyon D. Murray D. (1993). Applying User Modeling to Human-Computer Interaction Design. Artif. Intell. Rev. 7 (3-4), 199–225. 10.1007/BF00849555

[B6] Bii P. K. Too J. K. Mukwa C. W. (2018). Teacher Attitude towards Use of Chatbots in Routine Teaching. Univers. J. Educ. Res.. 6 (7), 1586–1597. 10.13189/ujer.2018.060719

[B7] Bii P. Too J. Langat R. (2013). An Investigation of Student’s Attitude Towards the Use of Chatbot Technology in Instruction: The Case of Knowie in a Selected High School. Education Research 4, 710–716. 10.14303/er.2013.231

[B8] Bos A. S. Pizzato M. C. Vettori M. Donato L. G. Soares P. P. Fagundes J. G. (2020). Empirical Evidence During the Implementation of an Educational Chatbot with the Electroencephalogram Metric. Creative Education 11, 2337–2345. 10.4236/CE.2020.1111171

[B9] Brusilovsky P. (2001). Adaptive Hypermedia. User Model. User-Adapted Interaction 11 (1), 87–110. 10.1023/a:1011143116306

[B10] Brusilovsky P. Millán E. (2007). “User Models for Adaptive Hypermedia and Adaptive Educational Systems,” in The Adaptive Web: Methods and Strategies of Web Personalization. Editors Brusilovsky P. Kobsa A. Nejdl W.. Berlin: Springer, 3–53. 10.1007/978-3-540-72079-9_1

[B11] Cabales V. (2019). “Muse: Scaffolding metacognitive reflection in design-based research,” in CHI EA’19: Extended Abstracts of the 2019 CHI Conference on Human Factors in Computing Systems, Glasgow, Scotland, United Kingdom, May 4–9, 2019, (ACM), 1–6. 10.1145/3290607.3308450

[B12] Carayannopoulos S. (2018). Using Chatbots to Aid Transition. Int. J. Info. Learn. Tech. 35, 118–129. 10.1108/IJILT-10-2017-0097

[B13] Chan C. H. Lee H. L. Lo W. K. Lui A. K.-F. (2018). Developing a Chatbot for College Student Programme Advisement. in 2018 International Symposium on Educational Technology, ISET 2018, Osaka, Japan, July 31–August 2, 2018. Editors Wang F. L. Iwasaki C. Konno T. Au O. Li C., (IEEE), 52–56. 10.1109/ISET.2018.00021

[B14] Chang M.-Y. Hwang J.-P. (2019). “Developing Chatbot with Deep Learning Techniques for Negotiation Course,” in 2019 8th International Congress on Advanced Applied Informatics, IIAI-AAI 2019, Toyama, Japan, July 7–11, 2019, (IEEE), 1047–1048. 10.1109/IIAI-AAI.2019.00220

[B15] Chen C.-A. Yang Y.-T. Wu S.-M. Chen H.-C. Chiu K.-C. Wu J.-W. (2018). “A Study of Implementing AI Chatbot in Campus Consulting Service”, in TANET 2018-Taiwan Internet Seminar, 1714–1719. 10.6861/TANET.201810.0317

[B16] Chen H.-L. Widarso G. V. Sutrisno H. (2020). A ChatBot for Learning Chinese: Learning Achievement and Technology Acceptance. J. Educ. Comput. Res. 58 (6), 1161–1189. 10.1177/0735633120929622

[B17] Daud S. H. M. Teo N. H. I. Zain N. H. M. (2020). E-java Chatbot for Learning Programming Language: A post-pandemic Alternative Virtual Tutor. Int. J. Emerging Trends Eng. Res. 8 (7). 3290–3298. 10.30534/ijeter/2020/67872020

[B18] Davies J. N. Verovko M. Verovko O. Solomakha I. (2020). “Personalization of E-Learning Process Using Ai-Powered Chatbot Integration,” in Selected Papers of 15th International Scientific-practical Conference, MODS, 2020: Advances in Intelligent Systems and Computing, Chernihiv, Ukraine, June 29–July 01, 2020. Editors Shkarlet S. Morozov A. Palagin A., (Springer) Vol. 1265, 209–216. 10.1007/978-3-030-58124-4_20

[B19] Diachenko A. V. Morgunov B. P. Melnyk T. P. Kravchenko O. I. Zubchenko L. V. (2019). The Use of Innovative Pedagogical Technologies for Automation of the Specialists' Professional Training. Int. J. Hydrogen. Energy. 8, 288–295. 10.5430/ijhe.v8n6p288

[B20] Dibitonto M. Leszczynska K. Tazzi F. Medaglia C. M. (2018). “Chatbot in a Campus Environment: Design of Lisa, a Virtual Assistant to Help Students in Their university Life,” in 20th International Conference, HCI International 2018, Las Vegas, NV, USA, July 15–20, 2018, Lecture Notes in Computer Science. Editors Kurosu M., (Springer), 103–116. 10.1007/978-3-319-91250-9

[B21] Durall E. Kapros E. (2020). “Co-design for a Competency Self-Assessment Chatbot and Survey in Science Education,” in 7th International Conference, LCT 2020, Held as Part of the 22nd HCI International Conference, HCII 2020, Copenhagen, Denmark, July 19–24, 2020, Lecture Notes in Computer Science. Editors Zaphiris P. Ioannou A., Berlin: Springer Vol. 12206, 13–23. 10.1007/978-3-030-50506-6_2

[B22] Duval E. Verbert K. (2012). Learning Analytics. Eleed 8 (1).

[B23] Engel J. D. Engel V. J. L. Mailoa E. (2020). Interaction Monitoring Model of Logo Counseling Website for College Students' Healthy Self-Esteem, I. J. Eval. Res. Educ. 9, 607–613. 10.11591/ijere.v9i3.20525

[B24] Febriani G. A. Agustia R. D. (2019). Development of Line Chatbot as a Learning Media for Mathematics National Exam Preparation. Elibrary.Unikom.Ac.Id. https://elibrary.unikom.ac.id/1130/14/UNIKOM_GISTY%20AMELIA%20FEBRIANI_JURNAL%20DALAM%20BAHASA%20INGGRIS.pdf.

[B25] Ferguson R. Sharples M. (2014). “Innovative Pedagogy at Massive Scale: Teaching and Learning in MOOCs,” in 9th European Conference on Technology Enhanced Learning, EC-TEL 2014, Graz, Austria, September 16–19, 2014, Lecture Notes in Computer Science. Editors Rensing C. de Freitas S. Ley T. Muñoz-Merino P. J., (Berlin: Springer) Vol. 8719, 98–111. 10.1007/978-3-319-11200-8_8

[B26] Fryer L. K. Ainley M. Thompson A. Gibson A. Sherlock Z. (2017). Stimulating and Sustaining Interest in a Language Course: An Experimental Comparison of Chatbot and Human Task Partners. Comput. Hum. Behav. 75, 461–468. 10.1016/j.chb.2017.05.045

[B27] Fryer L. K. Nakao K. Thompson A. (2019). Chatbot Learning Partners: Connecting Learning Experiences, Interest and Competence. Comput. Hum. Behav. 93, 279–289. 10.1016/j.chb.2018.12.023

[B28] Fryer L. K. Thompson A. Nakao K. Howarth M. Gallacher A. (2020). Supporting Self-Efficacy Beliefs and Interest as Educational Inputs and Outcomes: Framing AI and Human Partnered Task Experiences. Learn. Individual Differences, 80. 10.1016/j.lindif.2020.101850

[B29] Gabrielli S. Rizzi S. Carbone S. Donisi V. (2020). A Chatbot-Based Coaching Intervention for Adolescents to Promote Life Skills: Pilot Study. JMIR Hum. Factors 7 (1). 10.2196/16762 PMC7055808 32130128

[B30] Galko L. Porubän J. Senko J. (2018). “Improving the User Experience of Electronic University Enrollment,” in 16th IEEE International Conference on Emerging eLearning Technologies and Applications, ICETA 2018, Stary Smokovec, Slovakia, Nov 15–16, 2018. Editors Jakab F., (Piscataway, NJ: IEEE), 179–184. 10.1109/ICETA.2018.8572054

[B31] Goda Y. Yamada M. Matsukawa H. Hata K. Yasunami S. (2014). Conversation with a Chatbot before an Online EFL Group Discussion and the Effects on Critical Thinking. J. Inf. Syst. Edu. 13, 1–7. 10.12937/EJSISE.13.1

[B32] Graesser A. C. VanLehn K. Rose C. P. Jordan P. W. Harter D. (2001). Intelligent Tutoring Systems with Conversational Dialogue. AI Mag. 22 (4), 39–51. 10.1609/aimag.v22i4.1591

[B33] Greller W. Drachsler H. (2012). Translating Learning into Numbers: A Generic Framework for Learning Analytics. J. Educ. Tech. Soc. 15 (3), 42–57. 10.2307/jeductechsoci.15.3.42

[B68] Haristiani N. Rifa’i M. M. Combining Chatbot and Social Media: Enhancing Personal Learning Environment (PLE) in Language Learning. Indonesian J Sci Tech. 5 (3), 487–506. 10.17509/ijost.v5i3.28687

[B34] Hattie J. Timperley H. (2007). The Power of Feedback. Rev. Educ. Res. 77 (1), 81–112. 10.3102/003465430298487

[B35] Hattie J. (2009). Visible Learning: A Synthesis of over 800 Meta-Analyses Relating to Achievement. Abingdon, UK: Routledge.

[B36] Heller B. Proctor M. Mah D. Jewell L. Cheung B. (2005). “Freudbot: An Investigation of Chatbot Technology in Distance Education,” in Proceedings of ED-MEDIA 2005–World Conference on Educational Multimedia, Hypermedia and Telecommunications, Montréal, Canada, June 27–July 2, 2005. Editors Kommers P. Richards G., (AACE), 3913–3918.

[B37] Heo J. Lee J. (2019). “CiSA: An Inclusive Chatbot Service for International Students and Academics,” in 21st International Conference on Human-Computer Interaction, HCII 2019: Communications in Computer and Information Science, Orlando, FL, USA, July 26–31, 2019. Editors Stephanidis C., (Springer) 11786, 153–167. 10.1007/978-3-030-30033-3

[B38] Hobert S. (2019a). “How Are You, Chatbot? Evaluating Chatbots in Educational Settings - Results of a Literature Review,” in 17. Fachtagung Bildungstechnologien, DELFI 2019 - 17th Conference on Education Technologies, DELFI 2019, Berlin, Germany, Sept 16–19, 2019. Editors Pinkwart N. Konert J., 259–270. 10.18420/delfi2019_289

[B39] Hobert S. Meyer von Wolff R. (2019). “Say Hello to Your New Automated Tutor - A Structured Literature Review on Pedagogical Conversational Agents,” in 14th International Conference on Wirtschaftsinformatik, Siegen, Germany, Feb 23–27, 2019. Editors Pipek V. Ludwig T., (AIS).

[B40] Hobert S. (2019b). Say Hello to ‘Coding Tutor’! Design and Evaluation of a Chatbot-Based Learning System Supporting Students to Learn to Program in International Conference on Information Systems (ICIS) 2019 Conference, Munich, Germany, Dec 15–18, 2019, AIS 2661, 1–17.

[B41] Hobert S. (2020). Small Talk Conversations and the Long-Term Use of Chatbots in Educational Settings ‐ Experiences from a Field Study in 3rd International Workshop on Chatbot Research and Design, CONVERSATIONS 2019, Amsterdam, Netherlands, November 19–20: Lecture Notes in Computer Science. Editors Folstad A. Araujo T. Papadopoulos S. Law E. Granmo O. Luger E. Brandtzaeg P., (Springer) 11970, 260–272. 10.1007/978-3-030-39540-7_18

[B42] Hsieh S.-W. (2011). Effects of Cognitive Styles on an MSN Virtual Learning Companion System as an Adjunct to Classroom Instructions. Edu. Tech. Society 2, 161–174.

[B43] Huang J.-X. Kwon O.-W. Lee K.-S. Kim Y.-K. (2018). Improve the Chatbot Performance for the DB-CALL System Using a Hybrid Method and a Domain Corpus in Future-proof CALL: language learning as exploration and encounters–short papers from EUROCALL 2018, Jyväskylä, Finland, Aug 22–25, 2018. Editors Taalas P. Jalkanen J. Bradley L. Thouësny S., (Research-publishing.net). 10.14705/rpnet.2018.26.820

[B44] Huang W. Hew K. F. Gonda D. E. (2019). Designing and Evaluating Three Chatbot-Enhanced Activities for a Flipped Graduate Course. Int. J. Mech. Engineer. Robotics. Research. 813–818. 10.18178/ijmerr.8.5.813-818

[B45] Ismail M. Ade-Ibijola A. (2019). “Lecturer's Apprentice: A Chatbot for Assisting Novice Programmers,”in Proceedings - 2019 International Multidisciplinary Information Technology and Engineering Conference (IMITEC), Vanderbijlpark, South Africa, (IEEE), 1–8. 10.1109/IMITEC45504.2019.9015857

[B46] Jia J. (2008). “Motivate the Learners to Practice English through Playing with Chatbot CSIEC,” in 3rd International Conference on Technologies for E-Learning and Digital Entertainment, Edutainment 2008, Nanjing, China, June 25–27, 2008, Lecture Notes in Computer Science, (Springer) 5093, 180–191. 10.1007/978-3-540-69736-7_20

[B47] Jia J. (2004). “The Study of the Application of a Keywords-Based Chatbot System on the Teaching of Foreign Languages,” in Proceedings of SITE 2004--Society for Information Technology and Teacher Education International Conference, Atlanta, Georgia, USA. Editors Ferdig R. Crawford C. Carlsen R. Davis N. Price J. Weber R. Willis D., (AACE), 1201–1207.

[B48] Jivet I. Scheffel M. Drachsler H. Specht M. (2017). “Awareness is not enough: Pitfalls of learning analytics dashboards in the educational practice,” in 12th European Conference on Technology Enhanced Learning, EC-TEL 2017, Tallinn, Estonia, September 12–15, 2017, Lecture Notes in ComputerScience. Editors Lavoué E. Drachsler H. Verbert K. Broisin J. Pérez-Sanagustín M., (Springer), 82–96. 10.1007/978-3-319-66610-5_7

[B49] Jung H. Lee J. Park C. (2020). Deriving Design Principles for Educational Chatbots from Empirical Studies on Human-Chatbot Interaction. J. Digit. Contents Society, 21, 487–493. 10.9728/dcs.2020.21.3.487

[B50] Kerly A. Bull S. (2006). “The Potential for Chatbots in Negotiated Learner Modelling: A Wizard-Of-Oz Study,” in 8th International Conference on Intelligent Tutoring Systems, ITS 2006, Jhongli, Taiwan, June 26–30, 2006, Lecture Notes in Computer Science. Editors Ikeda M. Ashley K. D. Chan T. W., (Springer) 4053, 443–452. 10.1007/11774303

[B51] Kerly A. Ellis R. Bull S. (2008). CALMsystem: A Conversational Agent for Learner Modelling. Knowledge-Based Syst. 21, 238–246. 10.1016/j.knosys.2007.11.015

[B52] Kerly A. Hall P. Bull S. (2007). Bringing Chatbots into Education: Towards Natural Language Negotiation of Open Learner Models. Knowledge-Based Syst., 20, 177–185. 10.1016/j.knosys.2006.11.014

[B53] Kumar M. N. Chandar P. C. L. Prasad A. V. Sumangali K. (2016). “Android Based Educational Chatbot for Visually Impaired People,” in 2016 IEEE International Conference on Computational Intelligence and Computing Research, Chennai, India, December 15–17, 2016, 1–4. 10.1109/ICCIC.2016.7919664

[B54] Lee K. Jo J. Kim J. Kang Y. (2019). Can Chatbots Help Reduce the Workload of Administrative Officers? - Implementing and Deploying FAQ Chatbot Service in a University in 21st International Conference on Human-Computer Interaction, HCII 2019: Communications in Computer and Information Science, Orlando, FL, USA, July 26–31, 2019. Editors Stephanidis C., (Springer) 1032, 348–354. 10.1007/978-3-030-23522-2

[B55] Lester J. C. Converse S. A. Kahler S. E. Barlow S. T. Stone B. A. Bhogal R. S. (1997). “The Persona Effect: Affective Impact of Animated Pedagogical Agents,” in Proceedings of the ACM SIGCHI Conference on Human factors in computing systems, Atlanta, Georgia, USA, March 22–27, 1997, (ACM), 359–366.

[B56] Liberati A. Altman D. G. Tetzlaff J. Mulrow C. Gøtzsche P. C. Ioannidis J. P. A. (2009). The PRISMA Statement for Reporting Systematic Reviews and Meta-Analyses of Studies that Evaluate Health Care Interventions: Explanation and Elaboration. J. Clin. Epidemiol. 62 (10), e1–e34. 10.1016/j.jclinepi.2009.06.006 19631507

[B57] Lin M. P.-C. Chang D. (2020). Enhancing Post-secondary Writers’ Writing Skills with a Chatbot. J. Educ. Tech. Soc. 23, 78–92. 10.2307/26915408

[B58] Lin Y.-H. Tsai T. (2019). “A Conversational Assistant on Mobile Devices for Primitive Learners of Computer Programming,” in TALE 2019 - 2019 IEEE International Conference on Engineering, Technology and Education, Yogyakarta, Indonesia, December 10–13, 2019, (IEEE), 1–4. 10.1109/TALE48000.2019.9226015

[B59] Linden A. Fenn J. (2003). Understanding Gartner’s Hype Cycles. Strategic Analysis Report No. R-20-1971 8. Stamford, CT: Gartner, Inc.

[B60] Liu Q. Huang J. Wu L. Zhu K. Ba S. (2020). CBET: Design and Evaluation of a Domain-specific Chatbot for mobile Learning. Univ. Access Inf. Soc., 19, 655–673. 10.1007/s10209-019-00666-x

[B61] Mamani J. R. C. Álamo Y. J. R. Aguirre J. A. A. Toledo E. E. G. (2019). “Cognitive Services to Improve User Experience in Searching for Academic Information Based on Chatbot,” in Proceedings of the 2019 IEEE 26th International Conference on Electronics, Electrical Engineering and Computing (INTERCON), Lima, Peru, August 12–14, 2019, (IEEE), 1–4. 10.1109/INTERCON.2019.8853572

[B62] Martín-Martín A. Orduna-Malea E. Thelwall M. Delgado López-Cózar E. (2018). Google Scholar, Web of Science, and Scopus: A Systematic Comparison of Citations in 252 Subject Categories. J. Informetrics 12 (4), 1160–1177. 10.1016/j.joi.2018.09.002

[B63] Matsuura S. Ishimura R. (2017). Chatbot and Dialogue Demonstration with a Humanoid Robot in the Lecture Class, in 11th International Conference on Universal Access in Human-Computer Interaction, UAHCI 2017, held as part of the 19th International Conference on Human-Computer Interaction, HCI 2017, Vancouver, Canada, July 9–14, 2017, Lecture Notes in Computer Science. Editors Antona M. Stephanidis C., (Springer) Vol. 10279, 233–246. 10.1007/978-3-319-58700-4

[B64] Matsuura S. Omokawa R. (2020). Being Aware of One’s Self in the Auto-Generated Chat with a Communication Robot in UAHCI 2020, 477–488. 10.1007/978-3-030-49282-3

[B65] McLoughlin C. Oliver R. (1998). Maximising the Language and Learning Link in Computer Learning Environments. Br. J. Educ. Tech. 29 (2), 125–136. 10.1111/1467-8535.00054

[B66] Mendoza S. Hernández-León M. Sánchez-Adame L. M. Rodríguez J. Decouchant D. Meneses-Viveros A. (2020). “Supporting Student-Teacher Interaction through a Chatbot,” in 7th International Conference, LCT 2020, Held as Part of the 22nd HCI International Conference, HCII 2020, Copenhagen, Denmark, July 19–24, 2020, Lecture Notes in Computer Science. Editors Zaphiris P. Ioannou A., (Springer) 12206, 93–107. 10.1007/978-3-030-50506-6

[B67] Meyer V. Wolff R. Nörtemann J. Hobert S. Schumann M. (2020). “Chatbots for the Information Acquisition at Universities ‐ A Student’s View on the Application Area,“in 3rd International Workshop on Chatbot Research and Design, CONVERSATIONS 2019, Amsterdam, Netherlands, November 19–20, Lecture Notes in Computer Science. Editors Folstad A. Araujo T. Papadopoulos S. Law E. Granmo O. Luger E. Brandtzaeg P., (Springer) 11970, 231–244. 10.1007/978-3-030-39540-7

[B69] Na-Young K. (2018c). A Study on Chatbots for Developing Korean College Students’ English Listening and Reading Skills. J. Digital Convergence 16. 19–26. 10.14400/JDC.2018.16.8.019

[B70] Na-Young K. (2019). A Study on the Use of Artificial Intelligence Chatbots for Improving English Grammar Skills. J. Digital Convergence 17, 37–46. 10.14400/JDC.2019.17.8.037

[B71] Na-Young K. (2018a). Chatbots and Korean EFL Students’ English Vocabulary Learning. J. Digital Convergence 16. 1–7. 10.14400/JDC.2018.16.2.001

[B72] Na-Young K. (2018b). Different Chat Modes of a Chatbot and EFL Students’ Writing Skills Development. 1225–4975. 10.16933/sfle.2017.32.1.263

[B73] Na-Young K. (2017). Effects of Different Types of Chatbots on EFL Learners’ Speaking Competence and Learner Perception. Cross-Cultural Studies 48, 223–252. 10.21049/ccs.2017.48.223

[B74] Nagata R. Hashiguchi T. Sadoun D. (2020). Is the Simplest Chatbot Effective in English Writing Learning Assistance?, in 16th International Conference of the Pacific Association for Computational Linguistics, PACLING, Hanoi, Vietnam, October 11–13, 2019, Communications in Computer and Information Science. Editors Nguyen L.-M. Tojo S. Phan X.-H. Hasida K., (Springer) Vol. 1215, 245–246. 10.1007/978-981-15-6168-9

[B75] Nelson T. O. Narens L. (1994). Why Investigate Metacognition. in Metakognition: Knowing About Knowing. Editors Metcalfe J. Shimamura P., (MIT Press) 13, 1–25.

[B76] Nghi T. T. Phuc T. H. Thang N. T. (2019). Applying Ai Chatbot for Teaching a Foreign Language: An Empirical Research. Int. J. Sci. Res. 8.

[B77] Ondas S. Pleva M. Hládek D. (2019). How Chatbots Can Be Involved in the Education Process. in ICETA 2019 - 17th IEEE International Conference on Emerging eLearning Technologies and Applications, Proceedings, Stary Smokovec, Slovakia, November 21–22, 2019. Editors Jakab F., (IEEE), 575–580. 10.1109/ICETA48886.2019.9040095

[B78] Pereira J. Fernández-Raga M. Osuna-Acedo S. Roura-Redondo M. Almazán-López O. Buldón-Olalla A. (2019). Promoting Learners' Voice Productions Using Chatbots as a Tool for Improving the Learning Process in a MOOC. Tech. Know Learn. 24, 545–565. 10.1007/s10758-019-09414-9

[B79] Pérez J. Q. Daradoumis T. Puig J. M. M. (2020). Rediscovering the Use of Chatbots in Education: A Systematic Literature Review. Comput. Appl. Eng. Educ. 28, 1549–1565. 10.1002/cae.22326

[B80] Pérez-Marín D. (2021). A Review of the Practical Applications of Pedagogic Conversational Agents to Be Used in School and University Classrooms. Digital 1 (1), 18–33. 10.3390/digital1010002

[B81] Pham X. L. Pham T. Nguyen Q. M. Nguyen T. H. Cao T. T. H. (2018). “Chatbot as an Intelligent Personal Assistant for mobile Language Learning,” in ACM International Conference Proceeding Series 10.1145/3291078.3291115

[B82] Quincey E. de. Briggs C. Kyriacou T. Waller R. (2019). “Student Centred Design of a Learning Analytics System,” in Proceedings of the 9th International Conference on Learning Analytics & Knowledge, Tempe Arizona, USA, March 4–8, 2019, (ACM), 353–362. 10.1145/3303772.3303793

[B83] Ram A. Prasad R. Khatri C. Venkatesh A. Gabriel R. Liu Q (2018). Conversational Ai: The Science behind the Alexa Prize, in 1st Proceedings of Alexa Prize (Alexa Prize 2017). ArXiv [Preprint]. Available at: https://arxiv.org/abs/1801.03604.

[B84] Rebaque-Rivas P. Gil-Rodríguez E. (2019). Adopting an Omnichannel Approach to Improve User Experience in Online Enrolment at an E-Learning University, in 21st International Conference on Human-Computer Interaction, HCII 2019: Communications in Computer and Information Science, Orlando, FL, USA, July 26–31, 2019. Editors Stephanidis C., (Springer), 115–122. 10.1007/978-3-030-23525-3

[B85] Robinson C. (2019). Impressions of Viability: How Current Enrollment Management Personnel And Former Students Perceive The Implementation of A Chatbot Focused On Student Financial Communication. Higher Education Doctoral Projects.2. https://aquila.usm.edu/highereddoctoralprojects/2.

[B86] Ruan S. Jiang L. Xu J. Tham B. J.-K. Qiu Z. Zhu Y. Murnane E. L. Brunskill E. Landay J. A. (2019). “QuizBot: A Dialogue-based Adaptive Learning System for Factual Knowledge,” in 2019 CHI Conference on Human Factors in Computing Systems, CHI 2019, Glasgow, Scotland, United Kingdom, May 4–9, 2019, (ACM), 1–13. 10.1145/3290605.3300587

[B87] Sandoval Z. V. (2018). Design and Implementation of a Chatbot in Online Higher Education Settings. Issues Inf. Syst. 19, 44–52. 10.48009/4.iis.2018.44-52

[B88] Sandu N. Gide E. (2019). “Adoption of AI-Chatbots to Enhance Student Learning Experience in Higher Education in india,” in 18th International Conference on Information Technology Based Higher Education and Training, Magdeburg, Germany, September 26–27, 2019, (IEEE), 1–5. 10.1109/ITHET46829.2019.8937382

[B89] Saygin A. P. Cicekli I. Akman V. (2000). Turing Test: 50 Years Later. Minds and Machines 10 (4), 463–518. 10.1023/A:1011288000451

[B90] Sinclair A. McCurdy K. Lucas C. G. Lopez A. Gaševic D. (2019). “Tutorbot Corpus: Evidence of Human-Agent Verbal Alignment in Second Language Learner Dialogues,” in EDM 2019 - Proceedings of the 12th International Conference on Educational Data Mining.

[B91] Smutny P. Schreiberova P. (2020). Chatbots for Learning: A Review of Educational Chatbots for the Facebook Messenger. Comput. Edu. 151, 103862. 10.1016/j.compedu.2020.103862

[B92] Song D. Rice M. Oh E. Y. (2019). Participation in Online Courses and Interaction with a Virtual Agent. Int. Rev. Res. Open. Dis. 20, 44–62. 10.19173/irrodl.v20i1.3998

[B93] Stapić Z. Horvat A. Vukovac D. P. (2020). Designing a Faculty Chatbot through User-Centered Design Approach, in 22nd International Conference on Human-Computer Interaction,HCII 2020, Copenhagen, Denmark, July 19–24, 2020, Lecture Notes in Computer Science. Editors Stephanidis C. Harris D. Li W. C. Schmorrow D. D. Fidopiastis C. M. Zaphiris P., (Springer), 472–484. 10.1007/978-3-030-60128-7

[B94] Subramaniam N. K. (2019). Teaching and Learning via Chatbots with Immersive and Machine Learning Capabilities. In International Conference on Education (ICE 2019) Proceedings, Kuala Lumpur, Malaysia, April 10–11, 2019. Editors Ali S. A. H. Subramaniam T. T. Yusof S. M., 145–156.

[B95] Sugondo A. F. Bahana R. (2019). “Chatbot as an Alternative Means to Access Online Information Systems,” in 3rd International Conference on Eco Engineering Development, ICEED 2019, Surakarta, Indonesia, November 13–14, 2019, IOP Conference Series: Earth and Environmental Science, (IOP Publishing) 426. 10.1088/1755-1315/426/1/012168

[B96] Suwannatee S. Suwanyangyuen A. (2019). “Reading Chatbot” Mahidol University Library and Knowledge Center Smart Assistant,” in Proceedings for the 2019 International Conference on Library and Information Science (ICLIS), Taipei, Taiwan, July 11–13, 2019.

[B97] Vaidyam A. N. Wisniewski H. Halamka J. D. Kashavan M. S. Torous J. B. (2019). Chatbots and Conversational Agents in Mental Health: A Review of the Psychiatric Landscape. Can. J. Psychiatry 64 (7), 456–464. 10.1177/0706743719828977 30897957 PMC6610568

[B98] Vijayakumar B. Höhn S. Schommer C. (2019). “Quizbot: Exploring Formative Feedback with Conversational Interfaces,” in 21st International Conference on Technology Enhanced Assessment, TEA 2018, Amsterdam, Netherlands, Dec 10-11, 2018. Editors Draaijer S. Joosten-ten B. D. Ras E., (Springer), 102–120. 10.1007/978-3-030-25264-9

[B99] Virtanen M. A. Haavisto E. Liikanen E. Kääriäinen M. (2018). Ubiquitous Learning Environments in Higher Education: A Scoping Literature Review. Educ. Inf. Technol. 23 (2), 985–998. 10.1007/s10639-017-9646-6

[B100] Wildman T. M. Magliaro S. G. Niles R. A. Niles J. A. (1992). Teacher Mentoring: An Analysis of Roles, Activities, and Conditions. J. Teach. Edu. 43 (3), 205–213. 10.1177/0022487192043003007

[B101] Wiley D. Edwards E. K. (2002). Online Self-Organizing Social Systems: The Decentralized Future of Online Learning. Q. Rev. Distance Edu. 3 (1), 33–46.

[B102] Winkler R. Soellner M. (2018). Unleashing the Potential of Chatbots in Education: A State-Of-The-Art Analysis. in Academy of Management Annual Meeting Proceedings 2018 2018 (1), 15903. 10.5465/AMBPP.2018.15903abstract

[B103] Winne P. H. Hadwin A. F. (2008). “The Weave of Motivation and Self-Regulated Learning,” in Motivation and Self-Regulated Learning: Theory, Research, and Applications. Editors Schunk D. H. Zimmerman B. J., (Mahwah, NJ: Lawrence Erlbaum Associates Publishers), 297–314.

[B104] Wisniewski B. Zierer K. Hattie J. (2019). The Power of Feedback Revisited: A Meta-Analysis of Educational Feedback Research. Front. Psychol. 10, 1664–1078. 10.3389/fpsyg.2019.03087 32038429 PMC6987456

[B105] Wolfbauer I. Pammer-Schindler V. Rose C. P. (2020). “Rebo Junior: Analysis of Dialogue Structure Quality for a Reflection Guidance Chatbot,” in Proceedings of the Impact Papers at EC-TEL 2020, co-located with the 15th European Conference on Technology-Enhanced Learning “Addressing global challenges and quality education” (EC-TEL 2020), Virtual, Sept 14–18, 2020. Editors Broos T. Farrell T., 1–14.

[B106] Xiao Z. Zhou M. X. Fu W.-T. (2019). “Who should be my teammates: Using a conversational agent to understand individuals and help teaming,” in IUI’19: Proceedings of the 24th International Conference on Intelligent User Interfaces, Marina del Ray, California, USA, March 17–20, 2019, (ACM), 437–447. 10.1145/3301275.3302264

[B107] Xu A. Liu Z. Guo Y. Sinha V. Akkiraju R. (2017). “A New Chatbot for Customer Service on Social media,” in Proceedings of the 2017 CHI conference on human factors in computing systems, Denver, Colorado, USA, May 6–11, 2017, ACM, 3506–3510. 10.1145/3025453.3025496

[B108] Yin J. Goh T.-T. Yang B. Xiaobin Y. (2020). Conversation Technology with Micro-learning: The Impact of Chatbot-Based Learning on Students' Learning Motivation and Performance. J. Educ. Comput. Res. 59, 154–177. 10.1177/0735633120952067

